# *Casearia sylvestris* Derivatives Inhibit Inflammation and Oxidative Activity and Promote Healing in Human Gingival Fibroblasts – An *In Vitro* Study

**DOI:** 10.3290/j.ohpd.c_2831

**Published:** 2026-09-29

**Authors:** Angélica Letícia Reis Pavanelli, Maria Eduarda Scordamaia Lopes, André Thiago Reis, Flávio Alexandre Carvalho, Sven Zalewski, André Gonzaga dos Santos, Sigrun Eick, Joni Augusto Cirelli, Andressa Vilas Boas Nogueira, James Deschner

**Affiliations:** a Angélica Letícia Reis Pavanelli Dentist, PhD Candidate in Periodontology, Department of Periodontology and Operative Dentistry, University Medical Center of the Johannes Gutenberg University, Mainz, Germany; Department of Diagnosis and Surgery, School of Dentistry at Araraquara – São Paulo State University (UNESP), Araraquara, São Paulo, Brazil. Conceptualisation, methodology, validation, formal analysis, investigation, data curation, writing original draft preparation, writing review and editing, visualisation.; b Maria Eduarda Scordamaia Lopes Dentist, Postdoctoral Researcher, Department of Diagnosis and Surgery, School of Dentistry at Araraquara – São Paulo State University (UNESP), Araraquara, São Paulo, Brazil. Validation, investigation, preparation of the original draft, reviewing and editing.; c André Thiago Reis Undergraduate Researcher in Pharmaceutical Sciences, Department of Drugs and Medicines, School of Pharmaceutical Sciences – São Paulo State University (UNESP), Araraquara, São Paulo, Brazil. Methodology, validation, investigation, preparation and writing of the original draft, reviewing and editing.; d Flávio Alexandre Carvalho Pharmacist, Department of Drugs and Medicines, School of Pharmaceutical Sciences – São Paulo State University (UNESP), Araraquara, São Paulo, Brazil. Methodology, validation, preparation and writing of the original draft, reviewing and editing.; e Sven Zalewski Pharmacist, MSc, PhD Researcher in Pharmaceutical Sciences, Department of Drugs and Medicines, School of Pharmaceutical Sciences – São Paulo State University (UNESP), Araraquara, São Paulo, Brazil. Methodology, validation, preparation and writing of the original draft, reviewing and editing.; f André Gonzaga dos Santos Pharmacist, Assistant Professor, Department of Drugs and Medicines, School of Pharmaceutical Sciences – São Paulo State University (UNESP), Araraquara, São Paulo, Brazil. Methodology, validation, resources, writing original draft preparation, writing review and editing.; g Sigrun Eick Dentist, Professor and Head of the Oral Microbiology Laboratory, Department of Periodontology, School of Dental Medicine, University of Bern, Bern, Switzerland. Methodology, validation, resources, preparation and writing of the original draft, reviewing and editing.; h Joni Augusto Cirelli Dentist, Associate Professor, Department of Diagnosis and Surgery, School of Dentistry at Araraquara – São Paulo State University (UNESP), Araraquara, São Paulo, Brazil. Conceptualisation, methodology, validation, formal analysis, resources, preparation and writing of the original draft, reviewing and editing.; i Andressa Vilas Boas Nogueira Dentist, Associate Researcher and Head of the Research Laboratory Department of Periodontology and Operative Dentistry, University Medical Center of the Johannes Gutenberg University, Mainz, Germany. Conceptualisation, methodology, validation, formal analysis, investigation, resources, data curation, writing original draft preparation, writing review and editing, visualisation, supervision, project administration.; j James Deschner Dentist, Professor and Head, Department of Periodontology and Operative Dentistry, University Medical Center of the Johannes Gutenberg University, Mainz, Germany. Conceptualisation, methodology, validation, formal analysis, resources, writing original draft preparation, writing review and editing, visualisation, supervision, project administration.

**Keywords:** Casearia sylvestris, gingival fibroblasts, inflammation, periodontal diseases

## Abstract

**Purpose:**

New promising adjuvant approaches for managing periodontal inflammation are emerging. *Casearia sylvestris*, a medicinal plant rich in diterpenes, has demonstrated anti-inflammatory, antioxidant, and reparative properties. The present study aimed to investigate the anti-inflammatory, antiproteolytic, and pro-healing effects of *C. sylvestris* leaf derivatives: ethanolic extract (extract), diterpene-enriched fraction (F2), and isolated casearin J (Cas J) on human gingival fibroblasts (HGF).

**Methods and Materials:**

HGF were exposed to *Escherichia coli* lipopolysaccharide (LPS) and treated with *C. sylvestris* derivatives. Transcription and protein levels of TNF-α, IL-8, MMP-1, and iNOS were assessed by real-time polymerase chain reaction (PCR) and ELISA, respectively. Nitric oxide (NO) levels were measured using the Griess reaction. NF-κB p65 nuclear translocation and ERK1 phosphorylation were analysed by immunofluorescence and immunoassay, respectively. Cell viability and scratch wound-healing assays were also performed.

**Results:**

*E. coli* LPS triggered a significant pro-inflammatory and proteolytic cellular response, characterised by increased TNF-α, IL-8, MMP-1, iNOS, and NO levels. Treatment with *C. sylvestris* derivatives significantly reduced the levels of these mediators, while also inhibiting NF-κB p65 translocation and ERK1 phosphorylation. All compounds were biocompatible and promoted cell migration and wound closure *in vitro*.

**Conclusion:**

These findings suggest that *C. sylvestris* leaf derivatives exert anti-inflammatory, antiproteolytic, and pro-healing effects, thus highlighting their potential as adjunctive phytotherapeutic agents for periodontal disease management.

Gingivitis is defined as gingival inflammation resulting from continuous dental biofilm accumulation at and apical to the gingival margin. It is characterised by bleeding on probing and absence of attachment loss.^46^ The transition from gingival health to gingivitis is reversible after treatment. However, if left untreated, gingivitis can progress to periodontitis. As such, gingivitis is considered an important risk factor and a prerequisite for periodontitis, which is a chronic inflammation that compromises the supporting tissues of the teeth, such as gingiva, periodontal ligament, root cementum, and alveolar bone.^22^ Its progression involves a complex interaction between the subgingival dysbiotic microbiota and an excessive or dysregulated host immune-inflammatory response, leading to tooth loss in advanced stages.^23,24,51
^


Furthermore, this chronic inflammatory burden is not confined to the oral cavity but has been extensively linked to a wide range of systemic diseases.^32^ Several pro- and anti-inflammatory mediators are actively involved in the pathogenesis of periodontal diseases. Pro-inflammatory cytokines, such as tumour necrosis factor alpha (TNF-α), play a central role in amplifying the inflammatory response and activating matrix metalloproteinases (MMPs).^22,23
^ The critical role of these local and circulating cytokines in driving the pathogenesis and progression of periodontal diseases has been thoroughly documented.^3^ Interleukin (IL)-8 mediates neutrophil chemotaxis, thereby enhancing local inflammatory activity, while MMP-1 is responsible for the degradation of extracellular matrix components, including type I and type III collagen. In addition, MMP-1 is highly expressed in inflamed periodontal tissues and contributes significantly to periodontal destruction.^22,51
^


The treatment of periodontal diseases aims to reduce the dysbiotic biofilm through subgingival mechanical instrumentation and, in more severe cases, surgical procedures.^18^ The adjuvant use of antimicrobial agents, such as chlorhexidine and antibiotics, is also recommended, especially in situations of severe or refractory cases.^18^ However, prolonged use of these medicines can be associated with numerous adverse effects, such as the development of bacterial resistance, oral dysbiosis, and systemic side effects such as gastrointestinal disorders.^18,49
^ Recent research in dentistry has focused on natural products with anti-inflammatory, antioxidant, and regenerative properties.^6,41,47,48
^ Bioactive compounds such as curcumin from *Curcuma longa* L., polyphenol from *Passiflora edulis* Sims, tanshinone from *Salvia miltiorrhiza* Bunge, and the CaneCPI-5 fraction from sugarcane extract have demonstrated potential in modulating periodontal inflammation.^6,10,19,37,55
^ These agents act primarily through inhibition of inflammatory signalling pathways such as nuclear factor kappa B (NF-κB) and mitogen-activated protein kinase (MAPK), reduction of pro-inflammatory cytokines (e.g. TNF-α, IL-1β), and promotion of tissue healing.

In this context, *Casearia sylvestris*, a native plant from Latin America and popularly known as ‘guaçatonga’ in Brazil, has attracted attention due to its wide range of phytotherapeutic applications.^9,49
^ It has a rich and diverse phytochemical profile and is particularly abundant in clerodane-type diterpenes, such as casearins and caseargrewiins, which have been associated with anti-inflammatory and cytoprotective effects.^5^ In addition, it contains significant amounts of flavonoids (quercetin, rutin and hesperidin) and phenolic acids, compounds known for their antioxidant and wound-healing properties.^8,13,14
^
*In vitro* and *in vivo* studies have demonstrated that *C. sylvestris* extracts can modulate the expression of inflammatory mediators, reduce oxidative stress, and promote tissue repair in different biological models.^16,43,56
^ In the oral health context, previous research using human gingival keratinocytes showed that *C. sylvestris* derivatives were able to attenuate the pro-inflammatory cellular response to lipopolysaccharide (LPS).^48^ These properties position *C. sylvestris* as a promising candidate for the prevention and treatment of periodontal diseases. However, it remains unclear whether *C. sylvestris* derivatives modulate the effects of LPS on human gingival fibroblasts (HGF), another key cellular component of the gingiva. Therefore, the present study aimed to investigate the anti-inflammatory, antiproteolytic, antioxidant, and wound-healing properties of *C. sylvestris* ethanolic extract (extract), as well as diterpene-enriched fraction (F2) and isolated casearin J (Cas J) in HGF exposed to LPS.

## Methods and Materials

### Cell Culture

HGF were isolated from healthy gingiva of patients who had undergone wisdom tooth extraction at the University Medical Center Mainz in accordance with the Declaration of Helsinki and approved by the Ethics Committee of the Medical Association of Rhineland-Palatinate in Mainz, Germany (no. 2018-13858) on 11/22/2018. Written informed consent was obtained. Cell cultures were maintained in Dulbecco’s Modified Eagle Medium (DMEM, Invitrogen, Karlsruhe, Germany) supplemented with 10% foetal bovine serum (FBS, Invitrogen), 100 U/ml penicillin, and 100 µg/ml streptomycin (Invitrogen) at 37°C in a humidified atmosphere with 5% CO_2_. For the assays, cells between passages 3 and 7 were seeded (50,000 cells/well) on six-well culture plates and cultured until reaching approximately 80% confluence. Twenty-four hours before treatment, the medium was replaced with DMEM containing 1% FBS to reduce basal stimuli. In order to mimic an inflammatory microenvironment *in vitro*, HGF were exposed to 1 µg/ml of *Escherichia coli* LPS (O111: B4, Sigma-Aldrich, St. Louis, MO, USA) or *Fusobacterium nucleatum* ATCC 25586 (MOI of 25:1) for 30 min. Both concentrations were previously established.^47,48
^ Briefly, *F. nucleatum* was initially pre-cultured on Schaedler agar plates (Oxoid, Basingstoke, UK) under anaerobic conditions for 48 h. Then, bacterial cells were suspended in phosphate-buffered saline (PBS) to an optical density at 660 nm of 1.0, corresponding to approximately 1.2 × 10^9^ bacterial cells/ml. This suspension was submitted twice to ultrasonication (160 W for 15 min), ensuring complete bacterial inactivation. After exposing HGF to LPS from *E. coli* or *F. nucleatum*, *C. sylvestris* bioactive compounds such as 1 µg/ml of *C. sylvestris* extract, 1 µg/ml of F2, and 50 µg/ml of Cas J were simultaneously added for 24 h and 48 h. Control groups remained untreated.

### Plant Material, Extraction, Purification, and Quantification of Cas J

Leaves of *C. sylvestris* var. sylvestris were collected from 10 specimens in June 2024 at the School of Pharmaceutical Sciences, São Paulo State University, Araraquara, SP, Brazil (21º 48’ 88’’ 3-21º 48’ 98’’ 9 S, 48º11’ 86’’ 1-48 12’ 13’’ 3 W). This study was registered under the code A9B40A7 of the Brazilian National System for the Management of Genetic Heritage and Associated Traditional Knowledge (SisGen). Leaves were dried in an air-circulating oven at 40°C for 72 h, powdered using a knife mill, and extracted (3 kg) by maceration with ethanol (3:15 w/v, 72 h) at 40°C. Ethanol was evaporated using an IKA DEST KV 05S3 evaporator, yielding 300 g (10.0% w/w) of the dry extract. To purify the extract, 62 g was subjected to solid-phase extraction in a glass column (20 × 10 cm) using silica gel (63-200 µm)/activated charcoal (1:1, w/w). Elution was performed with hexane:ethyl acetate 95:05 (fraction 1-F1), ethyl acetate (fraction 2-F2), and methanol (fraction 3-F3). Subsequently, F2 (12.6 g) was submitted to silica gel column chromatography (20 × 5 cm, 40–63 µm), eluted with 1.3 L of hexane:ethyl acetate:isopropanol gradient (85:14:01; 78:20.5:1.5; 76:22.4:1.6; 73:25.2:1.8; 68:29.9:2.1, and 60:37.2:2.8), and 38 subfractions were collected. Cas J was purified from subfractions F2.11–12 using preparative HPLC-PDA/UV (Shimadzu® Corporation, Kyoto, Japan) equipped with an Agilent Eclipse XDB HPLC-PDA/UV C18 column (250 × 21.2 mm, 7 µm). Elution was performed using methanol 65% for 30 min, 1.8 ml injection volume, 15 ml/min flow rate, and monitored at 235 nm to yield 84.0 mg of Cas J (tR: 14.1 min; 98.3% purity). The chemical composition of the extract was identified by HPLC-MS, and Cas J was identified and quantified based on spectrometric data, as previously described.^8,48
^ Cas J content in the extract and in fraction F2 was 0.2% and 3.9%, respectively, whereas the total casearin content was 11.9% in the extract and 58.4% in fraction F2. In the extract, in addition to Cas J, other casearins were identified, including casearin B (Cas B; tR: 16.72 min), caseargrewiin F (casg F; tR: 14.02 min), and casearin N or O (f N or O; tR: 20.29 min).

### Real-Time PCR

Total RNA extraction was performed using the RNeasy Protect Mini Kit (Qiagen, Hilden, Germany) and its purity/concentration was verified by spectrophotometry (NanoDrop ND-2000, Thermo Fisher Scientific, Waltham, MA, USA). Synthesis of cDNA was performed using 500 ng of RNA and the iScript™ Select cDNA Synthesis kit (Bio-Rad Laboratories, Munich, Germany), according to the manufacturer’s protocol. PCR reactions were prepared using SYBR Green PCR Master Mix (SsoAdvanced Universal SYBR Green Supermix, Bio-Rad Laboratories) and specific primers (QuantiTect Primer Assay, Qiagen). Gene expression of TNF-α, IL-8, MMP-1, and inducible nitric oxide synthase (iNOS) was analysed using glyceraldehyde-3-phosphate dehydrogenase (GAPDH) as a reference gene. The reactions were carried out in a PCR thermal cycler (CFX96, Bio-Rad Laboratories) with a standard 40-cycle protocol, and the data were processed by the ΔΔCt method. The experiment was performed in triplicate and repeated three times.

### ELISA

The protein levels of TNF-α, IL-8 and MMP-1 in cell supernatants were measured using ELISA kits (DuoSet Human ELISA Kit, R&D Systems, Minneapolis, MN, USA), according to the manufacturer’s protocol. Absorbance readings were measured at 450 nm and optical correction at 540 nm using the BioTek Synergy H1 multimode reader (Agilent, Santa Clara, CA, USA). Cell numbers were controlled and there was no significant difference between groups.

### Nitrite Analysis

HGF were cultured and treated as previously described. After 24 h and 48 h, cell supernatants were collected and subjected to Griess reaction for quantification of nitrite (indirect indicator of nitric oxide (NO)). The Nitrite Assay Kit (Griess Reagent, MAK367, Sigma-Aldrich) was used following the manufacturer’s protocol. Supernatants were collected and incubated with reagents I and II on a 96-well plate for 10 min. The absorbance was measured at 540 nm using a multimode reader (BioTek Synergy H1, Agilent). Concentrations were calculated based on a standard curve. Assays were performed in duplicate and repeated in three independent experiments.

### Immunofluorescence

HGF cells were cultured in 24-well plates and treated as described above for up to 60 min. Afterwards, cells were washed with PBS, fixed with 4% paraformaldehyde for 10 min, and permeabilised with 0.1% Triton X-100 for 5 min. Blocking was performed with non-fat dry milk for 1 h. Samples were then incubated with primary anti-NF-κB p65 antibody (1:400, D14E12, Cell Signaling Technology) and then with secondary antibody conjugated to the CY3 fluorophore (1:2000, ab6939, Abcam, Cambridge, MA, USA). Fluorescence was visualised with the ZOE™ Fluorescence Cell Imager (Bio-Rad Laboratories). Untreated cells served as a control. Experiments were performed in duplicate and repeated in three independent experiments.

### Phospho-ERK1 Immunoassay

Detection of phosphorylated ERK1 was performed using the Lumit® Immunoassay Cellular System (CS3397A03, Promega Corporation, Madison, WI, USA), following the manufacturer’s instructions. HGF were cultured in 96-well plates (50,000 cells/well, Lumitrac, Greiner Bio-One GmbH, Kremsmünster, Austria) as described above. Cell treatment was performed in 40 µl of medium containing* C. sylvestris* extract, F2 or Cas J for 30 min followed by the addition of LPS for another 30 min. Next, 10 µl of lysis solution, prepared with Lumit® Lysis Buffer II and supplemented with protease and phosphatase inhibitors (Roche Diagnostics, Mannheim, Germany), was added to the cells. The plates were incubated at room temperature and shaken for 40 min. Then, 50 µl of antibody mix containing two primary antibodies against phospho-ERK (15 ng/well) and two Lumit secondary antibodies (0.3 µl each/well) were added to the cell lysates, followed by incubation at room temperature for 90 min. To assess the reagent background level and to calculate net bioluminescence signal, a set of cell lysate samples were treated with 50 μl of an antibody mix containing only the Lumit® secondary antibodies. Subsequently, 25 µl of Lumit® Detection Reagent was added to each well, and the plates were shaken for 2 min. After this period, luminescence was immediately measured in a multimode reader (BioTek Synergy H1, Agilent). The data obtained were expressed in relative luminescence units (RLU). Untreated cells served as the control. The experiment was performed in duplicate and repeated in three independent experiments.

### Cell Viability

Viability was analysed using the alamarBlue® assay (Invitrogen), based on the conversion of resazurin to resorufin, following the manufacturer’s protocol. HGF were cultured in 24-well plates (5,000 cells/well) for 24 h. Next, HGF were exposed to *E. coli* LPS or to different concentrations of *C. sylvestris* derivatives. After 24 h and 48 h, the medium was replaced with a solution containing 10% alamarBlue and incubated for 4 h at 37°C. Then, 100 µl of the medium was transferred to a 96-well plate, and the absorbance was measured at 570/600 nm using a multimode reader (BioTek Synergy H1, Agilent). Results were expressed as a percentage of reagent reduction in relation to the control. Assays were performed in duplicate and repeated in three independent experiments.

### Wound Healing

An *in vitro* wound-healing model using an automated cell scratch system was performed as previously published.^47,48
^ Briefly, HGF were cultured in 24-well plates (65,000 cells/well) until confluence. The wound was generated with the AutoScratch Wound Making Tool (Agilent) followed by three washing steps with medium to remove non-adherent cells and residues. Cells were then treated according to the experimental groups, as described above. Untreated cells were used as a control. Every 2 h, high-resolution kinetic images were acquired using the Lionheart FX Automated Microscope (Agilent) for 48 h. Quantitative analysis was performed using the Scratch Assay App (Agilent), which provides data on wound closure percentage and cellular wound repopulation rate. Experiments were repeated in three independent experiments.

### Statistical Analysis

Data were analysed using GraphPad Prism software (v.9.5.0; GraphPad Software, San Diego, CA, USA). Results were presented as mean ± SEM. Data normality was assessed using the Shapiro-Wilk test. Homogeneity of variances was evaluated using the Brown-Forsythe test. When variance heterogeneity was detected (*p* < 0.05), Welch’s one-way ANOVA was applied. Multiple comparisons were performed using Dunnett’s post hoc test, with the untreated control group as reference. Exact *p* values are reported in the Results section. Differences were considered statistically significant at *p* < 0.05.

## Results

### Effects of *C. sylvestris* Derivatives on LPS-Stimulated TNF-α, IL-8, and MMP-1

First, we investigated the regulation of TNF-α (Fig 1), IL-8 (Fig 2), and MMP-1 (Fig 3) in HGF exposed to *E. coli* LPS, with and without the addition of *C. sylvestris* extract, F2 or Cas J, at 24 h and 48 h. As analysed by real-time PCR, *E. coli* LPS alone significantly (*p* < 0.0001) upregulated the expression levels of TNF-α (Fig 1a), IL-8 (Fig 2a), and MMP-1 (Fig 3a) at both time points. However, these *E. coli* LPS-induced effects on TNF-α, IL-8, and MMP-1 were significantly (*p* < 0.0001) abrogated by *C. sylvestris* derivatives at 24 h and 48 h.

**Fig 1a to d Fig1atod:**
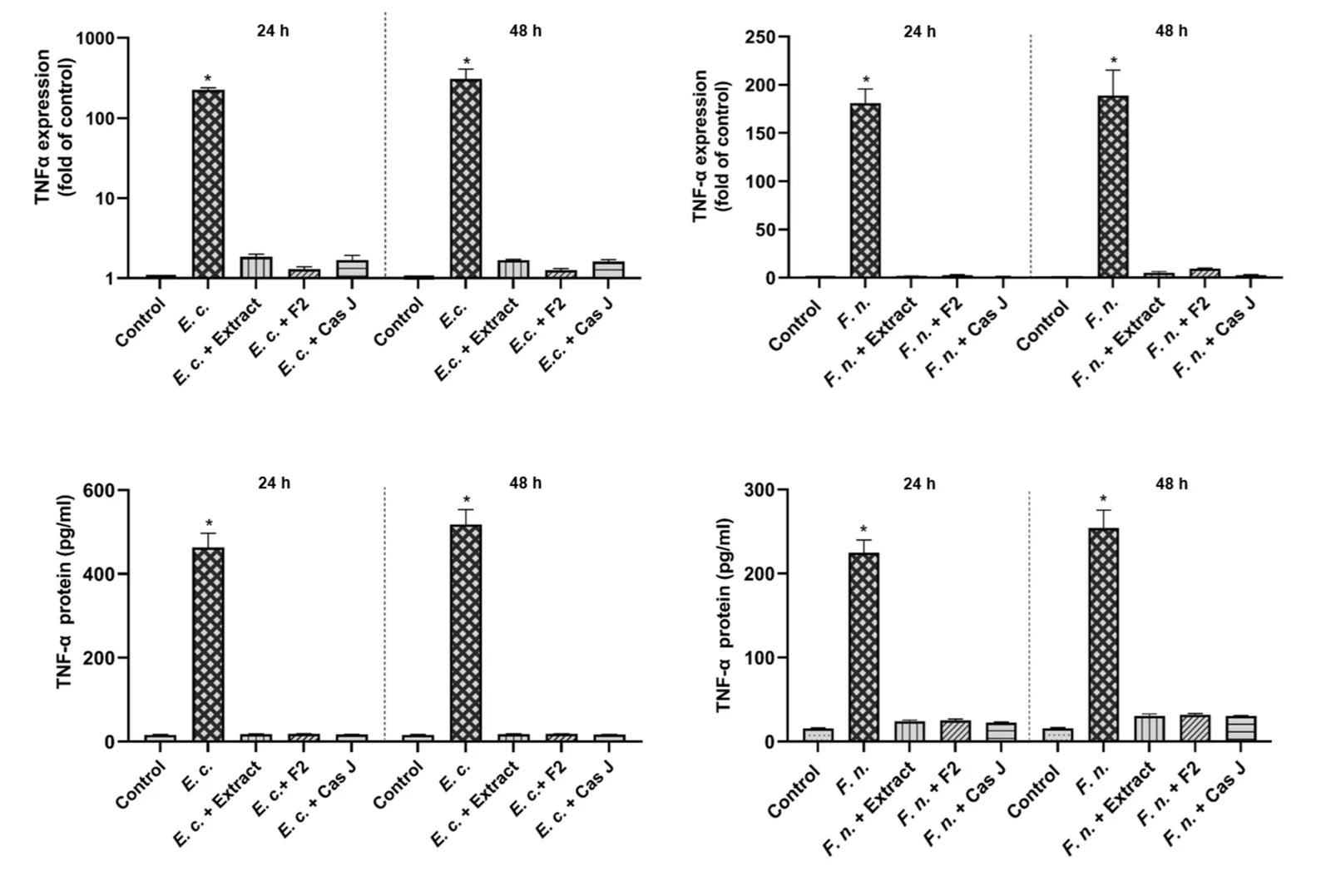
Effects of *C. sylvestris* and its derivatives on gene expression (a, c) and protein levels (b, d) of TNF-α in HGF. Cells were exposed to LPS from *E. coli* (*E. c.*) or *F. nucleatum* (*F. n*.) for 30 min and/or 1 µg/ml of *C. sylvestris* leaf derivatives ethanolic extract (extract), 1 µg/ml of *C. sylvestris* diterpene-enriched fraction (F2), and 50 µg/ml of isolated casearin J (Cas J) for 24 h and 48 h. The control group remained untreated. Data are presented as mean ± standard error of the mean (SEM, n = 9). Statistical analysis was performed by one-way ANOVA followed by Dunnett’s post-hoc test. * Significant (*p* < 0.0001) difference compared to all other groups.

**Fig 2a to d Fig2atod:**
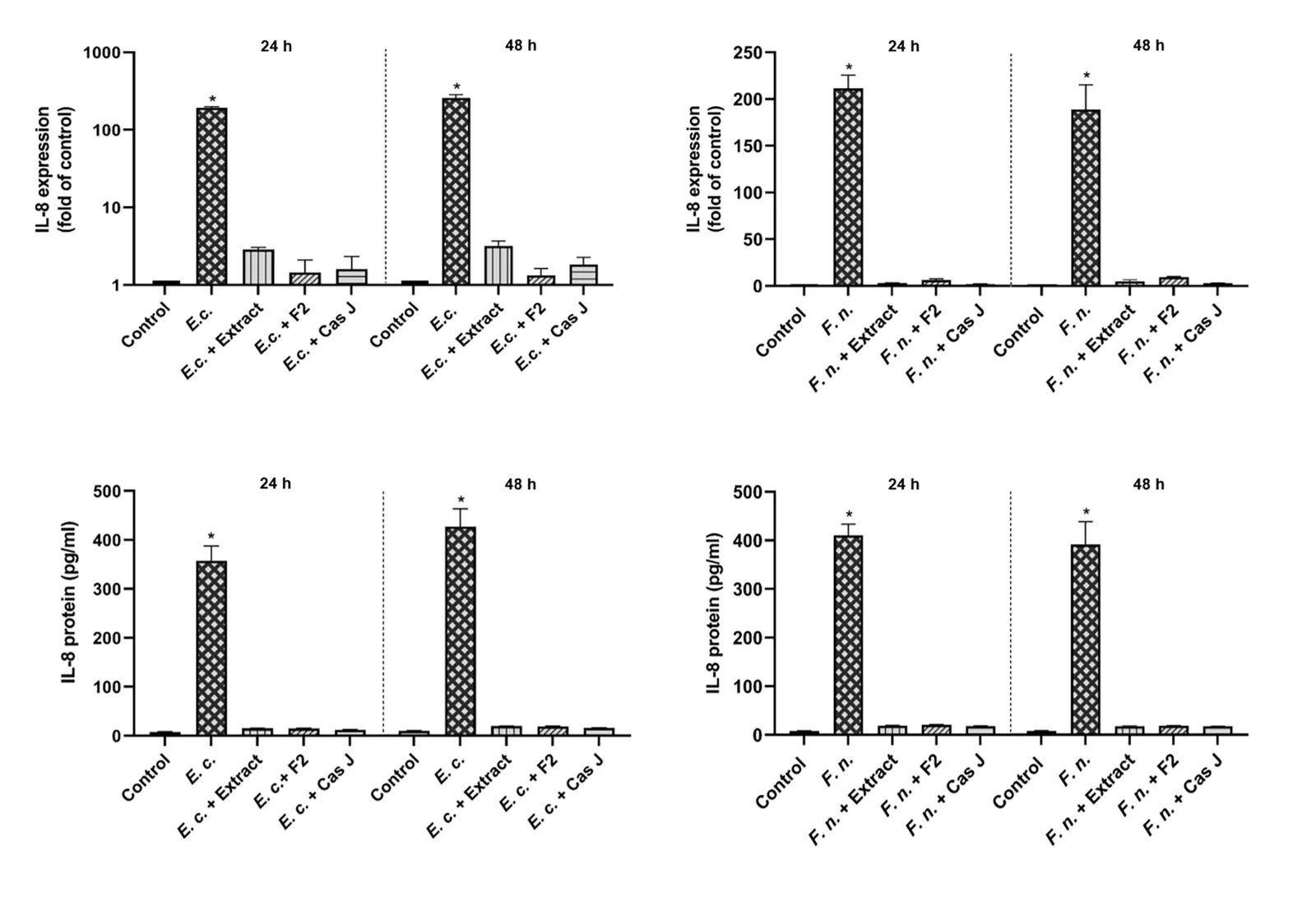
Effects of* C. sylvestris* and its derivatives on gene expression (a, c) and protein levels (b, d) of IL-8 in HGF. Cells were exposed to LPS from *E. coli* (*E. c.*) or *F. nucleatum* (*F. n.*) for 30 min and/or 1 µg/ml of *C. sylvestris* leaf derivatives ethanolic extract (extract), 1 µg/ml of *C. sylvestris* diterpene-enriched fraction (F2), and 50 µg/ml of isolated casearin J (Cas J) for 24 h and 48 h. The control group remained untreated. Data are presented as mean ± standard error of the mean (SEM, n = 9). Statistical analysis was performed by one-way ANOVA followed by Dunnett’s post-hoc test. * Significant (*p* < 0.0001) difference compared to all other groups.

**Fig 3a to d Fig3atod:**
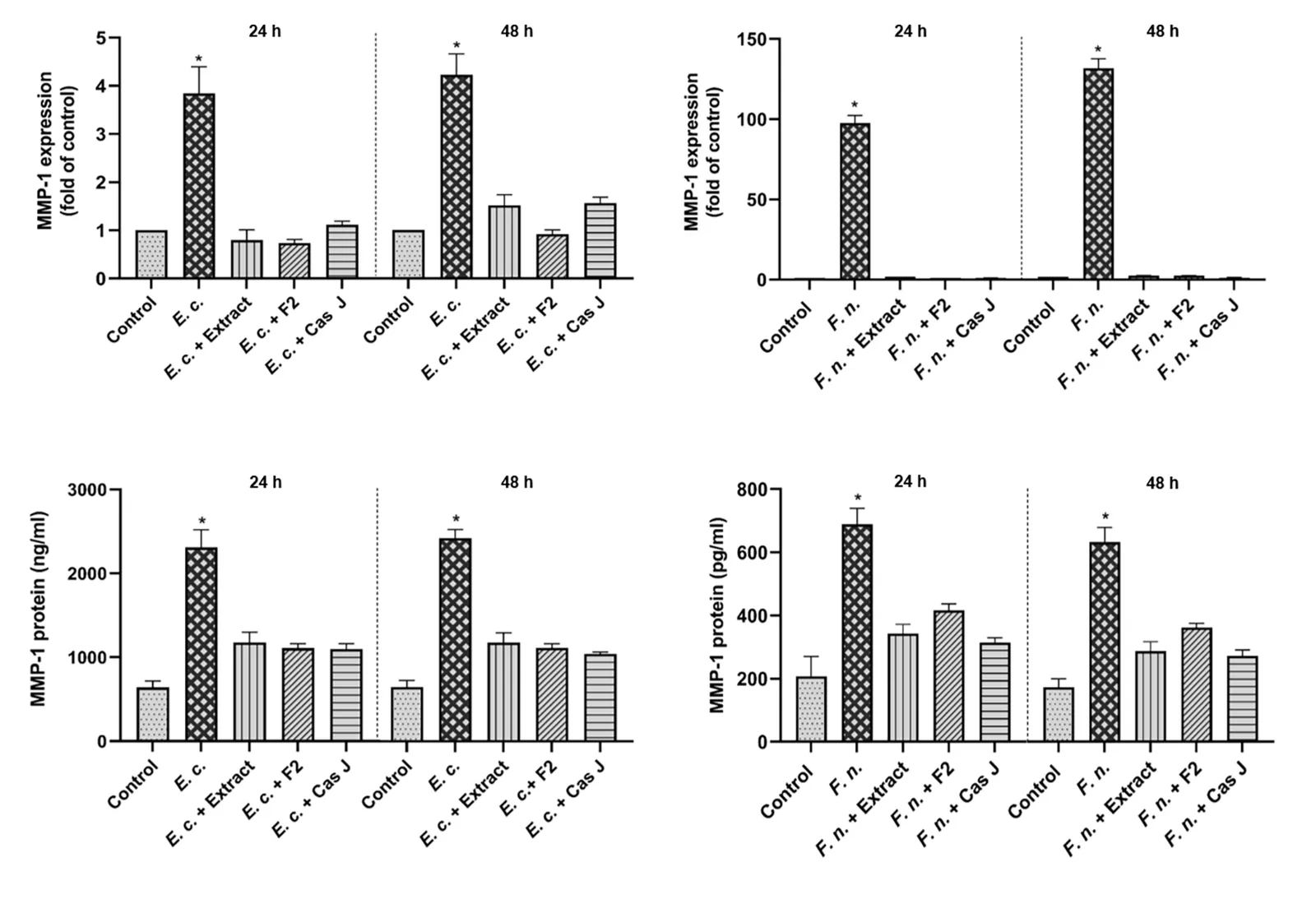
Effects of *C. sylvestris* and its derivatives on gene expression (a, c) and protein levels (b, d) of MMP-1 in HGF. Cells were exposed to LPS from *E. coli* (*E. c.*) or *F. nucleatum* (*F. n.*) for 30 min and/or 1 µg/ml of *C. sylvestris* leaf derivatives ethanolic extract (extract), 1 µg/ml of *C. sylvestris* diterpene-enriched fraction (F2), and 50 µg/ml of isolated casearin J (Cas J) for 24 h and 48 h. The control group remained untreated. Data are presented as mean ± standard error of the mean (SEM, n = 9). Statistical analysis was performed by one-way ANOVA followed by Dunnett’s post-hoc test. * Significant (*p* < 0.0001) difference compared to all other groups.

These modulatory effects were also observed at the protein level in cell culture supernatants, as measured by ELISA. Significantly enhanced (*p* < 0.0001) levels of TNF-α (Fig 1b), IL-8 (Fig 2b) and MMP-1 (Fig 3b) were observed in HGF challenged with *E. coli* LPS. Again, *C. sylvestris* derivatives were found to significantly (*p* < 0.0001) reverse these stimulatory effects of *E. coli* LPS.

Furthermore, we sought to determine whether LPS from the periodontal pathogen *F. nucleatum* would elicit effects similar to those of *E. coli*. HGF exposed to *F. nucleatum* LPS alone showed significantly (*p* < 0.0001) increased gene and protein levels of TNF-α (Fig 1c and d), IL-8 (Fig 2c and d), and MMP-1 (Fig 3c and d) at 24 h and 48 h. In the presence of *C. sylvestris* derivatives, the *F. nucleatum* LPS-stimulated effects on these molecules were significantly (*p* < 0.0001) counteracted.

### Influence of *C. sylvestris* Derivatives on LPS-Induced iNOS Gene Expression and NO

We subsequently evaluated the impact of *C. sylvestris* derivatives on iNOS expression by real-time PCR and NO using the Griess reaction at 24 h and 48 h. Exposure to *E. coli* LPS resulted in significant (*p* < 0.0001) upregulation of iNOS expression and a significant (*p* < 0.0001) increase in NO levels at both time points (Table 1). However, the *C. sylvestris* extract, F2, and Cas J significantly (*p* < 0.0001) inhibited the stimulatory actions of *E. coli* LPS on iNOS and NO at both time points (Table 1).

**Table 1 Table1:** Gene expression of iNOS and NO production measured by nitrite concentration in HGF exposed to *E. coli* LPS for 30 min and/or *C. sylvestris* derivatives for 24 h and 48 h. Untreated cells served as the control. Data are presented as mean ± standard error of the mean (SEM, n = 9)

Groups	24 h		48 h	
	iNOS expression (fold of control)	Nitrite concentration (µM)	iNOS expression (fold of control)	Nitrite concentration (µM)
Control	1.00	0.60	1.00	0.70
*E. c.*	6.04^1^	5.80^1^	10.73^1^	6.00^1^
*E. c.* + extract	2.00	2.00	1.19	1.50
*E. c.* + F2	0.79	1.90	0.87	1.40
*E. c.* + Cas J	1.89	1.70	1.26	1.30 ^1^
^1^Significant (*p* < 0.0001) difference compared to all other groups. E. c. (*E. coli*), extract (*C. sylvestris* leaf derivatives ethanolic extract), F2 (*C. sylvestris* diterpene-enriched fraction), and Cas J (isolated casearin J).				

### Impact of *C. sylvestris* Derivatives on LPS-Triggered NF-κB Nuclear Translocation and ERK1 Phosphorylation Level

To better understand how *C. sylvestris* derivatives modulate the effects of *E. coli* LPS intracellularly, the NF-κB and MAPK/ERK signalling pathways were investigated. As shown by immunofluorescence microscopy images (Fig 4a to e), the nuclear translocation of NF-κB p65 was triggered in many cells by exposure to *E. coli* LPS at 60 min, while this *E. coli* LPS-induced NF-κB p65 nuclear translocation was reduced by *C. sylvestris* derivatives.

**Fig 4a to f Fig4atof:**
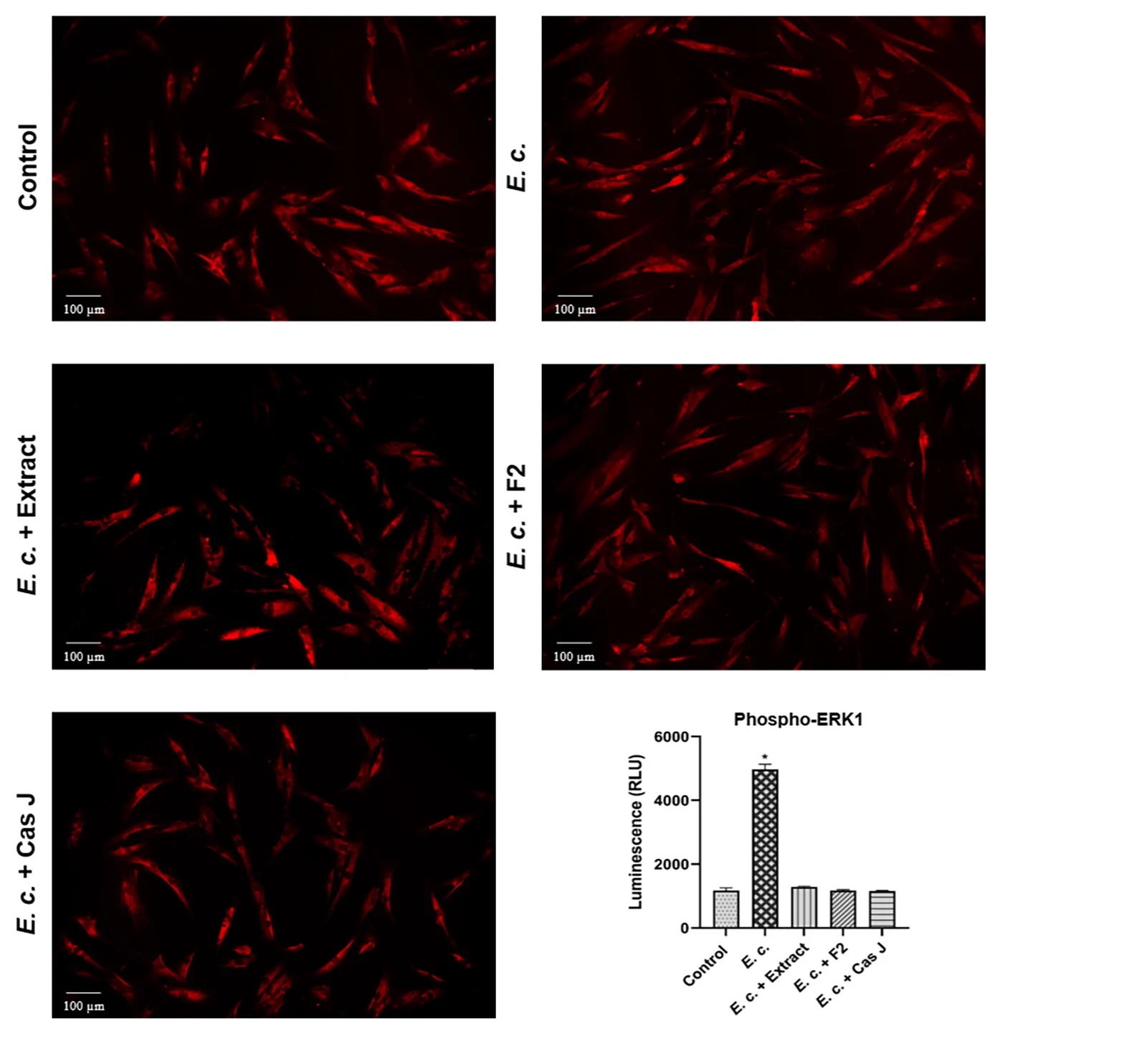
Signalling pathways investigated in HGF. NF-κB p65 nuclear translocation in HGF exposed to *E. coli* LPS for 30 min and/or *C. sylvestris* derivatives for 60 min, as evaluated by immunofluorescence microscopy. Untreated cells served as the control. Representative images from one set of experiments are presented (a to e). Activation of the MAPK/ERK signalling pathway in HGF exposed to *E. coli* LPS for 30 min and/or *C. sylvestris* derivatives for 30 min, as evaluated by phospho-ERK1 immunoassay. Untreated cells served as the control (f). Data are expressed in relative luminescence units (RLU) and presented as mean ± standard error of the mean (SEM, n = 9). Statistical analysis was performed by one-way ANOVA, followed by Dunnett’s post-hoc test. * Significant (*p* < 0.0001) difference compared to all other groups. *E. c.* (*E. coli*), extract (*C. sylvestris* leaf derivatives ethanolic extract), F2 (*C. sylvestris* diterpene-enriched fraction), and Cas J (isolated casearin J).

Additionally, *E. coli* LPS significantly (*p* < 0.0001) activated the MAPK/ERK signalling pathway by phosphorylation of extracellular signal-regulated kinase 1 (phospho-ERK1) at 30 min. The simultaneous treatment of *E. coli* LPS-stimulated HGF with *C. sylvestris* extract, F2 or Cas J resulted in a significant (*p* < 0.0001) reduction of phospho-ERK1 levels (Fig 4f).

### Effects of *E. coli* LPS and *C. sylvestris* Derivatives on Cell Viability

Next, we studied the viability of HGF exposed to *E. coli* LPS or varying concentrations of *C. sylvestris* derivatives. *E. coli* LPS caused a significant (*p* < 0.0001) decrease in cell viability by 35% at 24 h and approximately 41% at 48 h compared to the control. In contrast, *C. sylvestris* derivatives, at the tested concentrations, had no or only a minor (less than 10%) effect on cell viability over 48 h (Table 2).

**Table 2 Table2:** Cell viability of HGF exposed to *E. coli* LPS or various concentrations of *C. sylvestris* extract (1 µg/ml or 2 µg/ml), F2 (1 µg/ml or 3 µg/ml), or Cas J (50 µg/ml or 100 µg/ml) for 24 h and 48 h. Untreated cells served as the control. Data are expressed as the mean percentage of viable cells relative to the control (n = 9)

Groups	Cell viability (%)	
	24 h	48 h
Control	98.33	98.33
*E. c.*	65.00^1^	59.33^1^
extract (1 µg/ml)	97.33	91.33
extract (2 µg/ml)	95.00	90.20
F2 (1 µg/ml)	97.33	91.32
F2 (3 µg/ml)	95.33	90.33
Cas J (50 µg/ml)	96.22	90.67
Cas J (100 µg/ml)	95.30	90.67
^1^Significant (*p* < 0.0001) difference compared to all other groups. *E. c.* (*E. coli*), extract (*C. sylvestris* leaf derivatives ethanolic extract), F2 (*C. sylvestris* diterpene-enriched fraction), and Cas J (isolated casearin J).		

### Actions of *E. coli* LPS and *C. sylvestris* Derivatives on *In Vitro* Wound Healing

Finally, the impact of *E. coli* LPS and/or *C. sylvestris* compounds on *in vitro* wound healing was evaluated. Exposure of wounded HGF monolayers to *E. coli* LPS resulted in a reduction of wound closure, as observed by microscopy (Fig 5a and b). Furthermore, the inhibitory effect of *E. coli* LPS on wound closure was also reflected as reduced cellular wound repopulation rate in the presence of *E. coli* (Table 3). However, the *C. sylvestris* derivatives reduced the *E. coli* LPS inhibitory actions on wound closure and cellular wound repopulation rate throughout the 48-h period (Fig 5a and b and Table 3).

**Fig 5a and b Fig5aandb:**
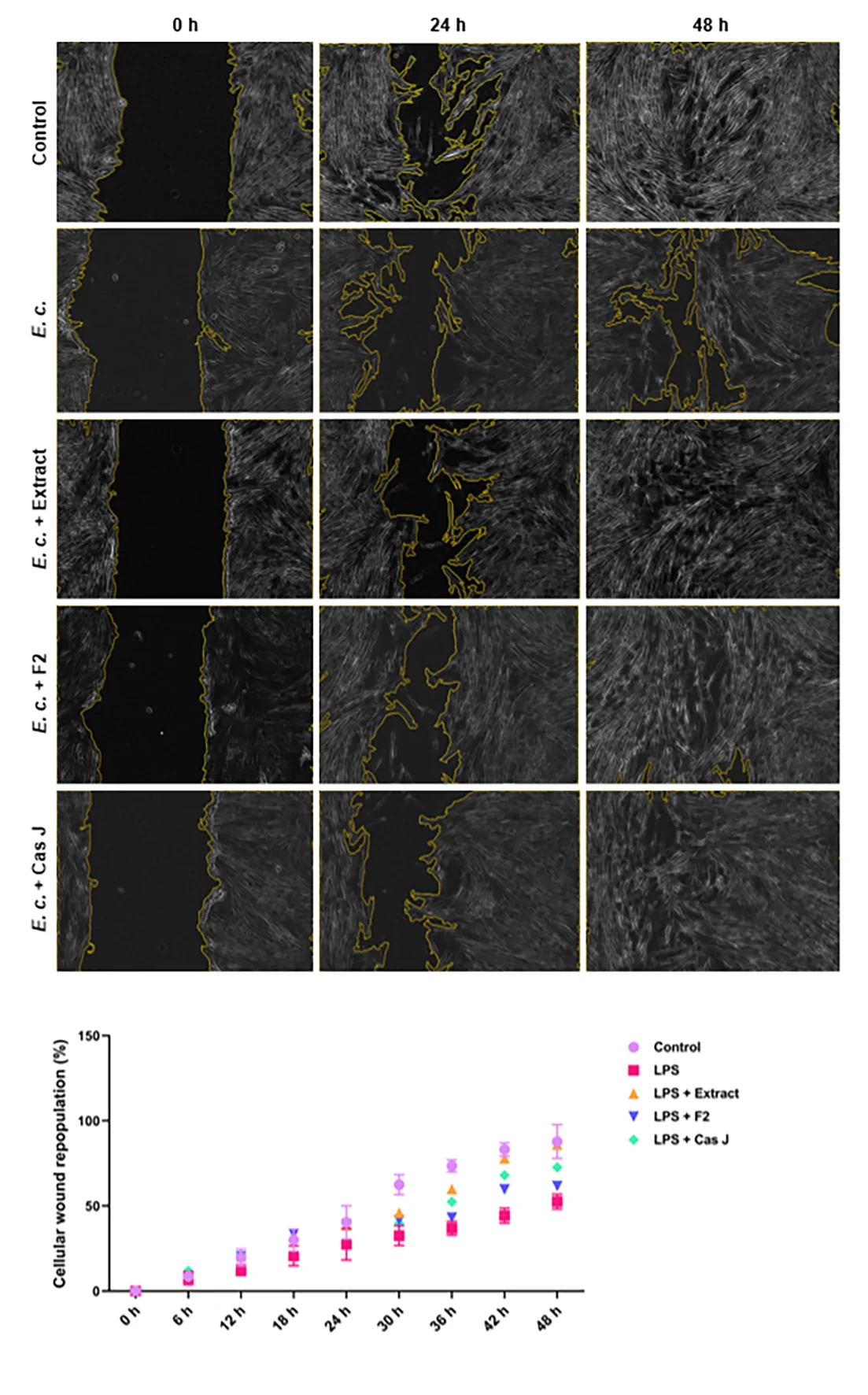
Wound closure in HGF monolayers. Cell monolayers were exposed to *E. coli* (*E. c*.) LPS for 30 min and/or 1 µg/ml of *C. sylvestris *leaf derivatives ethanolic extract (extract), 1 µg/ml of *C. sylvestris* diterpene-enriched fraction (F2), and 50 µg/ml of isolated casearin J (Cas J) for 48 h, as evaluated by high-contrast brightfield microscopy (a, b). Untreated monolayers served as a control. Representative images from one set of experiments are shown (a). Values are expressed as mean ± standard error of the mean (SEM, n = 9).

**Table 3 Table3:** Cellular wound repopulation rate (μm^2^/h) of HGF monolayers exposed to *E. coli* LPS for 30 min and/or *C. sylvestris* derivatives for 48 h, as evaluated by high-contrast brightfield microscopy. Untreated monolayers served as a control. Data are presented as mean ± standard error of the mean (SEM, n = 9)

Groups	Cellular wound repopulation rate (µm^2^/h)
Control	175.20
*E. c.*	92.50^1^
*E. c.* + extract	167.30
*E. c.* + F2	137.50
*E. c.* + Cas J	170.30
^1^Significant (*p* < 0.0001) difference compared to all other groups. *E. c.* (*E. coli*), extract (*C. sylvestris* leaf derivatives ethanolic extract), F2 (*C. sylvestris* diterpene-enriched fraction), and Cas J (isolated casearin J).	

## Discussion

In the present study, *C. sylvestris* leaf derivatives significantly attenuated pro-inflammatory responses of LPS-exposed gingival fibroblasts through modulation of NF-κB and ERK1 pathways, without compromising cell viability and while promoting healing. These findings corroborate the therapeutic potential of *C. sylvestris* as an adjuvant phytotherapeutic agent for periodontal disease management. To the best of our knowledge, this is the first study to assess the effects of *C. sylvestris* derivatives on HGF.

Over the past decade, the use of phytotherapeutic agents as adjunctive therapies for periodontal diseases has gained increasing scientific interest.^4,30,35
^ Recent reviews highlight that plant-derived extracts with anti-inflammatory, antimicrobial, and antioxidant properties may enhance the outcomes of conventional scaling and root planing.^30,35
^ Furthermore, studies have demonstrated that species such as Azadirachta indica, Camellia sinensis, and Magnolia kobus exert beneficial effects on gingivitis and periodontitis by reducing plaque index, tissue inflammation, and biomarkers associated with extracellular matrix degradation.^4,29,36,38
^ These findings underscore the relevance of exploring additional plant species with therapeutic potential, particularly within *in vitro* periodontal cell models.

*C. sylvestris*, a medicinal plant widely distributed in South and Central America, has attracted attention due to its rich phytochemical profile and broad pharmacological activities.^8,16,20
^ Its leaves contain bioactive compounds such as clerodane diterpenes, flavonoids, and essential oils, which have been associated with anti-inflammatory, antioxidant, wound healing, and cytoprotective properties.^8,9,20
^ Different derivatives from *C. sylvestris*, including crude ethanolic extracts, semi-purified fractions, and isolated diterpenes, have demonstrated the ability to modulate inflammatory mediators, inhibit oxidative stress, and promote tissue repair in various *in vitro* and *in vivo* models.^48,49
^ Our results on the chemical composition of the *C. sylvestris* leaf extract were consistent with data reported in the scientific literature.^8,56
^ These biological activities support the rationale for investigating *C. sylvestris* in oral and periodontal contexts, where inflammation, oxidative imbalance, and tissue destruction play key roles in disease progression.

Periodontal disease is characterised by inflammation driven by a dysregulated host response, in which the excessive production of pro-inflammatory cytokines, chemokines, matrix-degrading enzymes, and reactive nitrogen species plays a central role in tissue destruction and disease progression.^22,46,51
^ In our study, exposure of HGF to* E. coli* LPS induced a significant inflammatory response, with marked upregulation of TNF-α, IL-8, and MMP-1, as detected by RT-qPCR and ELISA. Importantly, these specific mediators are increasingly recognised as key biomarkers with high clinical utility for monitoring oral diseases non-invasively, such as through saliva.^15^ This elevated synthesis is highly relevant to periodontal disease pathogenesis, as TNF-α and IL-8 promote leukocyte recruitment, while MMP-1 contributes to extracellular matrix degradation by cleaving collagen.^22^ LPS stimulation also elevated iNOS gene expression and NO production, further reinforcing the inflammatory and oxidative profile, given that increased iNOS/NO activity is associated with enhanced oxidative stress, neutrophil activation, vasodilation, and connective tissue damage in periodontal tissues.^31,44
^ However, it is important to acknowledge that nitric oxide metabolism involves complex host-microbial interactions, playing a dual role in periodontal health that includes both microbial killing and immunoregulation.^52^


Treatment with *C. sylvestris* derivatives significantly attenuated this pro-inflammatory and pro-proteolytic environment, reducing the expression and/or production of TNF-α, IL-8, MMP-1, as well as iNOS and NO levels. This broad modulatory effect suggests that *C. sylvestris* suppresses key pathways involved in cytokine production, chemotactic signalling, matrix degradation, and oxidative imbalance. These findings are consistent with previous studies on *C. sylvestris* and other phytotherapeutic agents, such as *Curcuma longa*, *Passiflora edulis*, and the sugarcane cystatin CaneCPI-5, which have also been shown to reduce inflammatory mediators, MMP activity, and cellular activation.^7,19,41,45
^ Thus, our results support the potential of *C. sylvestris* as an adjuvant strategy for the prevention and management of periodontal diseases through its multifaceted anti-inflammatory, antioxidant, and matrix-protective effects.

The NF-κB pathway is considered central to the pathogenesis of periodontitis, regulating cytokines, chemokines, MMPs, and adhesion molecules, while the MAPK/ERK1 cascade is involved in cell proliferation, migration, and bacterial response.^57^ The modulation of these signalling axes by phytochemicals has emerged as a promising therapeutic strategy.^40,47,48,56,57
^ LPS is well known to activate both NF-κB and MAPK/ERK signalling pathways through the engagement of Toll-like receptor 4 (TLR4), triggering a cascade of intracellular events that amplify the inflammatory response^17,26,40
^; this classical activation pattern was also observed in our study upon HGF stimulation with *E. coli* LPS. However, these results with *C. sylvestris* derivatives suggest an inhibition of NF-κB p65 nuclear translocation and a reduction in ERK1 phosphorylation in LPS-stimulated HGF. This dual suppression of key inflammatory pathways is consistent with the significant reduction we observed in cytokine and MMP-1 expression. Interestingly, *Magnolia kobus* extracts inhibited NF-κB and MAPK activation in *Porphyromonas gingivalis* LPS-stimulated macrophages,^38^ and similar anti-inflammatory modulation has been described for other medicinal plants in oral inflammation models.^17,26,37,40
^


Furthermore, although modulation of the MAPK/ERK pathway by *C. sylvestris* derivatives has been previously reported in a distinct biological context, this evidence was limited to tumour cells and other isolated compounds.^21^ Ferreira-Silva et al^21^ demonstrated that casearin D inhibited ERK phosphorylation and downregulated cyclin D1 in hepatocellular carcinoma cells (HepG2), highlighting a potential interaction between clerodane diterpenes and ERK signalling outside inflammatory models. However, no studies to date have investigated ERK modulation by *C. sylvestris* derivatives in primary periodontal cells or under inflammatory conditions relevant to periodontal disease. Therefore, the present findings represent the first demonstration that *C. sylvestris* derivatives attenuate ERK1 phosphorylation in LPS-stimulated HGF, extending previous observations to a physiologically relevant, non-tumoural, connective tissue-derived cell model and supporting a broader mechanistic role for these compounds in periodontal inflammation and tissue repair.

The biocompatibility analysis demonstrated that although *E. coli* LPS stimulation reduced HGF viability, treatment with *C. sylvestris* derivatives did not significantly affect cellular viability. The absence of toxicity is a prerequisite for considering therapeutic applications and supports the progression to wound-healing and preclinical models.^11,34,54
^ In addition to the viability assessment, we also investigated the effects of *C. sylvestris* in an *in vitro* wound-healing assay. In this analysis, LPS markedly delayed fibroblast migration and repopulation of the wound area, demonstrating its detrimental impact on tissue repair. However, treatment with *C. sylvestris* derivatives effectively neutralised this negative influence, restoring wound closure and enhancing cell migration. Taken together with the finding that *C. sylvestris* does not reduce cell viability, these results suggest that the extract may promote wound-healing under inflammatory conditions, which has translational relevance for clinical periodontal regeneration. This effect is highly relevant to periodontics since regenerative procedures rely on fibroblast migration, matrix remodelling, and efficient repopulation.^1,24,53
^ Similar results have been reported in the literature on medicinal plants for oral wound-healing, where passion fruit and curcumin extracts improved fibroblast or epithelial wound closure *in vitro*.^1,47,53
^ Taken together, these results reinforce the potential application of *C. sylvestris* as an adjunctive therapy in gingival and peri-implant tissue regeneration.

The methodological design of this study relied on primary HGF, a widely accepted cellular model for investigating host-pathogen interactions and inflammatory mechanisms relevant to periodontal tissues. As an inflammatory stimulus, we employed purified *E. coli* LPS, which is commonly used in biomedical research due to its commercial availability, structural consistency, and well-characterised ability to trigger TLR4-mediated pro-inflammatory signalling.^39,48
^ Although *E. coli* LPS is not a classical periodontopathogen-derived endotoxin, several studies have demonstrated that its effects closely resemble those induced by LPS from *F. nucleatum*, a key bridging organism in subgingival biofilms, particularly regarding the upregulation of cytokines, chemokines, and matrix-degrading enzymes, as well as activation of NF-κB and MAPK pathways.^33,40,42,59
^ Similar inflammatory and proteolytic responses have also been reported for LPS from other periodontopathogens, including *P. gingivalis* and *Aggregatibacter actinomycetemcomitans*.^2,12,42,50
^


It is important to note, however, that periodontitis is not driven solely by LPS. Other bacterial virulence factors such as proteases (e.g. gingipains), fimbriae, outer membrane vesicles, and metabolic byproducts play fundamental roles in tissue destruction and immune modulation.^25,58,59
^ Moreover, the pathogenic potential of subgingival biofilms arises from the combined action of multiple microorganisms rather than a single species. Therefore, although the present model using *E. coli* LPS provides a controlled and reproducible system for evaluating the anti-inflammatory and wound-healing potential of *C. sylvestris*, future studies should incorporate more complex stimuli, including LPS from different periodontopathogens, multispecies biofilms, and additional virulence factors, to better replicate the multifactorial nature of periodontal disease.^27,28
^


Some limitations of this study should be acknowledged. The *in vitro* HGF model does not fully replicate the tissue complexity of the periodontium, which involves epithelial, immune, and bone cells as well as microbial interactions. Furthermore, it is essential to investigate the effects of bioactive compounds and new drugs across different periodontal cell types. Given that each cell population possesses distinct functional roles within the periodontal environment, understanding their specific mechanisms in response to novel treatments is crucial for developing more targeted and effective therapeutic strategies. The demonstration that *C. sylvestris* derivatives can simultaneously suppress pro-inflammatory signalling (NF-κB/ERK) and enhance wound repopulation in HGF is a critical step towards defining its clinical potential. HGF are not merely structural components; they are central orchestrators of the periodontium’s immune-inflammatory response and matrix remodelling.^54^ Therefore, the observed reduction in IL-8 and MMP-1 levels suggests a potential mechanism for limiting neutrophil recruitment and collagen degradation within the periodontal tissues.^22,51
^ However, the clinical relevance of these results must be viewed through the lens of periodontitis as a systemic and polymicrobial disease. Recent evidence emphasises that the success of any adjunctive phytotherapy depends on its ability to function within a dysbiotic environment where biomarkers, such as those analysed here, serve as vital indicators of both local destruction and systemic burden.^3,15
^ Moreover, the role of NO modulation by *C. sylvestris* deserves careful clinical attention, as NO metabolism is a key player in the delicate balance between microbial killing and tissue protection.^52^ While our study establishes a robust mechanistic foundation, the transition to clinical application requires human trials to evaluate the safety and efficacy of targeted delivery systems (e.g. local gels or nanoparticles) in mitigating the excessive host response while maintaining homeostatic oral health.^15^ Despite these limitations, the present findings contribute to advancing the understanding of phytotherapeutic compounds in periodontal modulation.

## Conclusion

In conclusion, *C. sylvestris* leaf derivatives significantly attenuated pro-inflammatory responses in LPS-exposed gingival fibroblasts and promoted wound healing under inflammatory conditions. These effects were accompanied by reduced NF-κB p65 nuclear translocation and ERK1 phosphorylation, suggesting a potential association of the NF-κB and MAPK/ERK signalling pathways. These results corroborate previous data from our group and reinforce the therapeutic potential of *C. sylvestris* as an adjuvant agent in periodontal therapy.

### Acknowledgements

We would like to thank São Paulo Research Foundation (FAPESP) for the scholarship grants to ALRP (2022/13096-5 and 2024/09363-3) and Antonietta Valentino for her valuable technical support. This research was partially funded by the Coordination for the Improvement of Higher Education Personnel – Brazil (CAPES) – Financing Code 001 and by the National Institute of Science and Technology of Biodiversity and Natural Products – INCT-BioNat (CNPq and FAPESP, grant number 465637/2014-0). Furthermore, the present research was supported by the University Medical Center of the Johannes Gutenberg University.

## References
